# Autonomy over authority: the role of autonomous motivation in law compliance

**DOI:** 10.1007/s11031-025-10155-9

**Published:** 2025-09-04

**Authors:** Anna Tovmasyan, Daria Onitiu, Sandra Wachter, Brent Mittelstadt, Netta Weinstein

**Affiliations:** 1 School of Psychology and Clinical Language Sciences, University of Reading, Reading, UK; 2 Oxford Internet Institute, University of Oxford, Oxford, UK; 3 Hasso Plattner Institute, Potsdam, Germany

**Keywords:** SDT, Motivation, Law, Values, Healthcare

## Abstract

Fear of punishment and perceived legitimacy of power are often believed to be key drivers of compliance with the law. Three studies challenged this view through the lens of Self-Determination Theory (SDT), which posits that motivation can reflect internal values alongside such external forces, and in doing so stretched SDT into an important domain in which societal principles may inadvertently undermine motivation. Participants evaluated a proposed healthcare data law, presented in a clinical context, that pitted data privacy against the goal of building inclusive AI systems. Autonomous motivation to follow the law was consistently associated with intended law compliance. Conversely, controlled motivation driven by expectations of consequences showed mixed (positive or absent) effects on intended compliance. These results emphasize that relying on the threat of punishment may be insufficient for ensuring law compliance. Laws must be written in a way that resonate with values held by the public.

Communities define right from wrong actions to maintain social order, with the onus falling on policymakers and governing bodies to construct and then enforce laws in ways that maximize public compliance to socially desirable action. At a societal level, the primary system serving this purpose is the law, a system that obliges individuals, through external controls, to act in pre-specified ways to avoid punishment delivered through fines, restrictions on behavior, or other consequences (Marshall, 1893). The current studies examined these widely accepted motivational antecedents of law compliance (in a form of *controlled* motivation), alongside a less utilized form– *autonomous* motivation– reflecting internal valuing, from the perspective of Self-Determination Theory (SDT; Deci & Ryan, 2000; Ryan & Deci, 2000).

## Perspectives on governance

Broadly speaking, a punitive approach to governance adopted in legal systems worldwide emphasizes the importance of establishing appropriate consequences to discourage individuals’ disobeying the law. The assumption underlying this approach is that individuals, when faced with the prospect of punishment, are more likely to conform to societal norms and legal regulations (Kahan, 1999; Nagin, 1998; Simpson et al., 2013). Research shows that in line with these perspectives in law, people obey a law because they fear punishments associated with failure to comply (Tyler, 2006). Modern approaches recognize more nuance and emphasize *justifying* the execution of power alongside utilizing deterrence and consequences (i.e., legitimacy; Tyler, 2006). For example, people accept police officers’ right to dictate appropriate behavior not only when they feel a duty to obey officers, but also when they believe that the institution acts according to a shared moral purpose with citizens (Jackson et al., 2012). Other work has found that at times, people may comply because a law is consistent with their values (Tyler, 2006), or in other words, because the law aligns with their core beliefs about what is fundamentally important (Maio, 2016).

## Self-determination perspectives on law compliance

Building on these new perspectives on motivating law compliance, we posit that a focus on punitive measures neglects the complex interplay of individual motivations and personal values that underlie how individuals understand and respond to laws. We test this view from a SDT (Ryan & Deci, 2000) perspective, which challenges the traditional assumption that fear of punishment is the sole or even the most effective motivator for driving behavior, including, in the current studies, legal compliance. SDT argues that the level of *internalization* of a given behavior (i.e., the extent to which motivation is taken in, or internalized, from the environment and into the self; Ryan & Deci, 2000) predicts cooperation with that behavior (Van Petegem et al., 2021). SDT further differentiates between two types of motivations that reflect high or low levels of internalization, respectively. The first of these– autonomous motivation– reflects motivation from within the self, including from one’s *personally held* values, interests, and core identity. Behavior that is autonomously motivated is felt to be volitional, choiceful, and self-endorsed. In contrast, controlled motivation is driven by external demands and pressures including punishments and coercion, as well as by self-enforced punishments of shame and guilt when failing to behave (Deci & Ryan, 2000).

Studies outside of law have shown that autonomous motivation predicts greater engagement in the motivated behavior (Koestner et al., 2008). For example, Steg (2016) reviewed studies showing that individuals who endorse pro-environmental values are more likely to reduce energy consumption and adopt eco-friendly transport. Similarly, students with autonomous academic motivation persist longer and perform better than those driven by external pressures (Koestner et al., 2008).

Expectations that internalization facilitates cooperation with the *law*, specifically, are supported by emerging research integrating SDT and procedural justice model perspectives (Van Petegem et al., 2021). The authors examined adolescents’ reasons for complying with the law by asking participants to respond to the prompt, “I obey the law because…” followed by items measuring autonomous motivation (e.g., “…I understand why this is important”) and controlled regulation (e.g., “…otherwise I will be punished”; adapted from Soenens et al., 2009). The study found that adolescents who better internalized legal norms had a higher intention to comply with those norms. The nascent literature on the psychology of law begs the question: is merely enacting a law enough to drive compliance, or does the quality of people’s motivation, namely, whether it is autonomous or controlled, play a role? The current paper explores this and by doing so addresses how motivation for the law and its underpinning values drive intention to comply with it.

## Value-action gap and value tension 

The solution of making values salient to increase law compliance faces some challenges. One important concern is the observation that a disconnect exists between having a value and behaving in line with it, a phenomenon known as the value-action gap (Tavri, 2021). This gap has been primarily observed in relation to environmental behaviors when individuals who endorse sustainability as a value fail to behave in ways that conserve the natural environment (Barr, 2006; Kennedy et al., 2009). In complex social systems where behavior has many influences, it may be the case that the values underpinning a behavior clash with opposing values held by the individual; that is, acting on one value entails acting against the other (i.e., there is a value tension; Hitlin & Piliavin, 2004). For example, in the context of law, an individual having pacifist beliefs may decide against joining the army, even if the law, underpinned by the value of protecting one’s immediate community, obliges them to do so. Thus, based on an individual’s value set, the decision to follow the law can create a gap between value and action.

To address this complexity, the current studies tested not only individuals’ motivation for the laws as broadly autonomous (i.e., from within the values of the self) or controlled– a common SDT operationalization (Ryan & Deci, 2017), but also the importance that individuals placed on the *specific values* that underly a law. Doing so recognizes potential nuance in motivation when behavior, including law compliance, is driven by multiple values that act on self-motivation concurrently.

We apply this principle to legal frameworks around emerging technologies such as healthcare, an area in which new laws are being formed and revised regularly (Pesapane et al., 2021), and where motivation qualities may play a key role. Such laws, historically, have been written to protect people’s data; those laws have given expression to the value of privacy, which recognizes that individuals deserve to choose on access and use of their personal information (Gerety, 1977). An example of such a law is the General Data Protection Regulation (GDPR), a European framework that protects personal data of individuals (Dove, 2018; Wachter & Mittelstadt, 2019). Yet, even in this example we can see where value tensions may arise: GDPR also adheres to a *different* value of inclusion, which is characterized by ensuring that data processing activities are “fair” (General Data Protection Regulation, Article 5).

If expressions this law could be modified to better express privacy, or inclusion, but not both, this dilemma poses: Would the individual choose to break the law and act in line with their value, or will they obey the law to avoid being punished? The current research therefore seeks to examine the behavioral mechanisms when values are in tension by applying SDT to this field and looking at its application in a complex “value collision” scenarios.

### Concurrent motivational drivers of behavior intention

A further challenge of applying SDT to the field of law is that, while most studies in SDT find that autonomous motivation better predicts behavior (Ryan & Deci, 2017), it is also plausible that motivating lawful behavior involves both controlled and autonomous forms of motivation. Previous research on rules compliance and moral decision-making demonstrates that individuals may choose to “hide behind the rules” to shed themselves of moral responsibilities (Kihl, 2007). In addition, Gaudreau et al. (2012) found that, although they heightened stress and reduced intrinsic interest over time, externally imposed deadlines still increased student task completion rates in the short term. Similarly, while they bred resentment, workplace policies enforcing compliance through threat of penalties initially led to higher compliance (Fast & Schnurr, 2021). These findings suggest that controlled motivation can elicit immediate compliance, although it comes with longer-term costs to well-being.

In addition, controlled motivation can conduce behavior when there is high legitimacy associated with enacting the behavior, as in the case of controlling parenting being associated with reduced cyberbullying by adolescents (Legate et al., 2019). Similarly, feeling choiceful appears to be less important for behavioral intention to reduce workplace prejudice, presumably because organizational restrictions on prejudiced behavior in the policing workplace is seen by staff and officers to be legitimate (Weinstein et al., 2023). In such cases, there is a question of how important motivations driven by choice and volition are when perceived legitimacy is highly salient. The law provides an intriguing extension to ask this question.

## The current research

This research examined the extent to which people’s motivations for complying with the law– specifically, their autonomous (value-driven) or controlled (driven by threat of external or internally imposed punishments) motivations– predict their intention to comply. Given that laws often reflect core societal values (Dror, 1957), it follows that individuals who autonomously internalize these values should be more likely to comply with legal mandates. It is plausible that people would be more likely to follow laws that are internalized and align with their values. If autonomous motivation is indeed associated with law compliance, this suggests that the existing in the legal field focus on controlled motivation (i.e., punishment) may not be the only way to ensure compliance. Such an application of SDT informs law and policymaking, highlighting the importance of writing laws that intentionally speak to desired values underlying desired behavior.

To examine these questions, we developed a proof-of-concept hypothetical law in the area of healthcare artificial intelligence (AI) and recruited healthcare professionals to test it. The law presented to participants revolved around sharing sensitive patient data, a topic that encapsulates competing values of inclusion (i.e., promoting equal access and use of data for innovation) and privacy (i.e., protecting patient confidentiality). Such an approach reflects the real-world complexity of legal decision-making, where healthcare professionals must balance competing ethical considerations. This tension mirrors broader societal debates around AI and data privacy (Gangadharan, 2017). All studies explored whether autonomous and controlled motivation to follow the law was associated with intended law compliance (i.e., behavioral intention to cooperate with the law) in a sample of healthcare professionals.

The current research therefore brings an SDT perspective to legal literatures, informing both in the following ways. First, this interdisciplinary work applies the psychological principles of SDT to a legal field and examines the motivational factors that have previously been ignored. Second, we apply SDT to the principles of value tension by examining how individuals navigate competing values and what drives decision making in legal scenarios. Third, we apply these SDT principles in the field of AI– an area of growing significance, where laws are still being shaped, making this study both timely and highly relevant. By integrating these perspectives, this research challenges conventional assumptions and paves the way for a more nuanced understanding of law compliance.

## Transparency and openness

We report how we determined our sample size, all data exclusions, manipulations, and measures in the study. The pilot study (Study 1) was not pre-registered. The design, methodology, hypotheses, and analyses of Studies 2 and 3 were pre-registered on the Open Science Framework (OSF; labelled "Study 2" and "Study 4 on OSF, respectively). All materials, data, analysis scripts, and supplementary materials are available on the OSF: https://osf.io/nsy92/?view_only=364335cf6b2645169ac616ef107cc113.

## Study 1

Study 1 was designed to explore links between autonomous and controlled motivation to follow the law and intention to comply with the law. Given the neglected role of motivation in communicating laws, exploring this link is critical. Should laws communicated to the public rely primarily on conveying controlled motivation such as threat of punishment or obligation? Or should they make salient meaningful and personally consequential reasons for the law? Prior research shows that autonomous motivation predicts behavioral engagement in multiple domains (Koestner et al., 2008; Van Petegem et al., 2021). Because compliance is more likely when people internalize and endorse the rationale for rules, we predicted that autonomous motivation would positively predict intention to comply with the proposed law. While some studies find that controlled motivation can drive behavior in high-legitimacy contexts (Legate et al., 2019), others suggest it is less effective long-term and may even reduce engagement over time (Fast & Schnurr, 2021; Gaudreau et al., 2012). Given that legal compliance is often framed in terms of external enforcement, we predicted that controlled motivation would also be associated with higher intent to comply.

Based on previous research, we hypothesized (H) the following:

### Hypothesis 1

Those higher on both autonomous and controlled motivation to follow the proposed law would have higher intent to follow this proposed law.

### Method

#### Participants

We recruited *N* = 185 healthcare professionals, aged 18 and over via Prolific.com. Of these, 22 were excluded for failing our attention check (described below), and four were excluded because they worked in animal but not human healthcare. The remaining 133 participants (81 women; 51 men; one non-binary) had a mean age of 35.36 years (*SD* = 12.20, range = 18–75 years). The sample was predominantly White (65%), 12% being Black or African American, 9% Asian, 1% American Indian or Alaskan Native, and 13% “Other”.

#### Materials

*Ethics*: For this and future studies, all participants were treated in accordance with American Psychological Association ethical guidelines for research (Sales & Folkman, 2000) and the World Medical Association Declaration of Helsinki (World Medical Association, 2013). The research was approved by the School of Psychology ethics committee at the University of Reading (2024-012-NW).

*Law description*: Participants were asked to read a proposed law newly constructed for the study in collaboration with two co-authors who are legal scholars in AI governance. Among other text, participants read:

“A new law has been proposed that will help to regulate fairness of healthcare artificial intelligence (AI). The law aims to ensure that all patients, independent of their race, gender, age, socioeconomic status, and other demographic variables, receive equally accurate AI-produced diagnosis and treatment plan. The consequence of this data gap is a skewed algorithmic understanding, potentially perpetuating disparities in healthcare outcomes. To reduce bias, the proposed law will oblige clinicians to share sensitive information about their patients. Hospitals and public health bodies would now be able to make patients' data– such as electronic health records - available for training an algorithm used for diagnosis. This broad spectrum of information aims to provide a more nuanced understanding of patients' health contexts, fostering the development of AI models that can better account for diverse influences on medical outcomes. This is a divergence from the current practices, which do not oblige clinicians to share full information about every patient. This law would allow AI algorithms to reduce bias, improve the development of individualized treatment and have better predictive abilities”.

Participants were asked to summarize the proposed law in their own words, which was used as a validation check of their understanding.

##### Predictor variables

*Reasons to follow the law*: We assessed motivation to follow the law by asking participants to complete the questionnaire on a 0 ("*strongly disagree"*) to 100 ("*strongly agree"*) assessing their reasons for following the law. Autonomous motivation was measured with the items: “Because I believe this law is fair, just, and legitimate”, “Because the ways that it benefits society are important to me”, and “Because acting in line with it is consistent with who I am”. Controlled motivation was measured with the items: “Because I don't want to be punished by law”, “Because I care about my reputation”. We have initially pre-registered the item “Because I would feel I have no choice” to be included in controlled motivation measure, but opted to subsequently remove it, following a reviewer suggestion, as it is more consistent with amotivation, rather than controlled motivation measure (the same approach was taken across all reported studies). Autonomous (McDonald’s ω =.88, 95% CI [.85,.92]) and controlled (*r* =.44, *p* <.001) motivation items were summed to create the “autonomous motivation” and “controlled motivation” variables.

*Values held*: Following previous research (Schwartz, 2006), we measured having values, in this case those underlying the proposed law, by asking them to rate their agreement on a scale from 0 (*not at all like me*) to a 100 (*exactly like me*) of whether they agree with the following: “How much are you like this person?”: “This person values inclusion” (assessing “inclusion” value), “This person values privacy” (assessing “privacy” value).

##### Outcome variable

*Intended law compliance*: In this study, intended law compliance was selected as an appropriate proxy for behavior intention given the law proposed could not be directly followed by our participants, as it was hypothetical. Participants were asked “Would you be willing to follow this law?” on a sliding scale from 0 to 100 that ranged from “*definitely not”* to *“definitely yes*”. This continuous scale allowed us to measure participants' likelihood of law compliance, providing richer variance in responses compared to a binary measure.

#### Attention and comprehension check

For the purposes of attention and comprehension check, participants were asked to check three out of five accurate statements. The statements were: “The law described above would be relevant to healthcare” (accurate), “The law described above would restrict patients’ use of data” (inaccurate), “The law described above would require sharing sensitive patient data” (accurate), “The law described above would inform more inclusive [less biased] healthcare technology” (accurate), and “The law described above would risk more biased [less inclusive] healthcare technology” (inaccurate). These items effectively identified participants who did not recall the law was about healthcare, in line with inclusion value, or in tension with privacy value.

### Results

#### Analytic strategy

Analysis was conducted in RStudio, Version 2023.06.0 + 421. We used multiple regression to test two predictors– autonomous and controlled motivations– simultaneously, with the outcome variable: intended law compliance. To isolate the effects of law motivation (specifically, autonomous and controlled motivations), we controlled for participants’ having the values of privacy and inclusion by adding them as predictors for the models. After the initial model was run, non-statistically significant predictors were removed from the model to improve model fit (using backward elimination method, see Heinze et al., 2018). Below, we present the results for both full and reduced models.

Intended law compliance was significantly and positively associated with autonomous motivation (*r* =.63, *p* <.001, large effect size) and valuing inclusion (*r* =.21, *p* <.05, small effect size). Additionally, autonomous motivation and valuing inclusion were positively correlated (*r* =.19, *p* <.05, small effect size), and valuing inclusion was positively correlated with controlled motivation (*r* =.18, *p* <.05, small effect size) and valuing privacy (*r* =.22, *p* <.05, small effect size). No other significant correlations were observed. See Table 1 for full details.

**Table 1:** Study 1 correlation between key variables

	1	2	3	4
1. Intended law compliance	–			
2. Autonomy for law	.63***	–		
3. Control for law	.06	−.01	–	
4. Valuing inclusion	.21*	.19*	.18*	–
5. Valuing privacy	.02	−.05	.14	.22*

*p <.05, **p <.01, ***p <.001. Autonomy = autonomous motivation (variable 2); Control = controlled motivation (variable 3)

Results for the full model are presented in Table 2. Only autonomous motivation to follow the law was a statistically significant predictor of intended law compliance. When non-statistically significant predictors were removed, results indicated that those higher on autonomous motivation (*b* =.20, *SE* =.02, *p* <.001) had greater compliance intention.

**Table 2:** Study 1 predictors of intended law compliance from the full model

Model	Predictor	*b*	*SE*	β	*t*	*p*
H_0_	Intercept	70.81	2.20		32.18	<.001
H_1_	Intercept	20.68	9.35		2.21	.029
	Autonomy for law	.20	.02	.62	8.95	<.001
	Control for law	.02	.03	.05	0.70	.486
	Valuing privacy	.10	.09	.08	1.14	.256
	Valuing inclusion	.02	.08	.02	.27	.789

### Brief discussion

Study 1 results indicated that autonomous, but not controlled, motivation was associated with intended law compliance, partially supporting Hypothesis 1, which anticipated that controlled motivation would relate to greater intended compliance. In addition, although participants who valued inclusion (the value that underlined the law) were more likely to intend to behave in line with the law, this relationship was no longer in evidence in models that controlled for autonomous motivation for the law. The privacy value that stood in contrast with the proposed law showed no relationship with intended compliance in either correlations or simultaneous models.

Effectively, these findings suggested a “value-action gap” (Ajzen & Fishbein, 1975; LaPiere, 1934): individuals’ values did not align with support for the laws that expressed them. They mirror previous research from law on the “privacy paradox” suggest that there is a discrepancy between users expressing concern over their privacy online and their actual behavior online (Barth & De Jong, 2017; Solove, 2021). In all, these findings support an SDT perspective positing that autonomous motivation underlies behavioral intention (Ryan & Deci, 2000), and questions conceptual views that values and laws interrelate (Dror, 1957).

## Study 2

Study 1 analyses controlled for having a law-consistent inclusion value and a law-inconsistent privacy value and found weak effects for their contributions to intention to follow the law. However, the relationship between values and behavioral intention may be more complicated than we recognized within our Study 1 approach. Presumably, values themselves can be held for autonomous reasons (e.g., because the value is personally important and tied into identity) or controlled reasons (e.g., out of a sense of obligation to have the value), and in those cases, *the more internalization that one has around their value*, the more they intend to behave in line with that value (Deci & Ryan, 2000).

Indeed, while *valuing* something, such as a law or a moral principle, is typically considered an expression of autonomous motivation, originating within the self (Deci & Ryan, 2000), values such as privacy or inclusion– those tested in Study 1– are abstract ideas which can be internalized to a greater or lesser extent. For example, researchers have identified that people can be more or less autonomous when they hold environmental values (see Steg, 2016, for a review) as well as when they make sustainable food choices based on those values (Schösler et al., 2014). Similarly, although not studied, legal scholars emphasize that laws reflect societal values and that the degree to which individuals endorse those values affects compliance (Quelle, 2018). If laws derive from values, then the extent to which people autonomously or controllingly endorse the values underlying laws may be key to predicting law compliance.

To address these possibilities, Study 2 replicated Study 1 and extended it with four new predictors: autonomous and controlled motivations to act in line with value of inclusion (the value consistent with the proposed law) and autonomous and controlled motivations to act in line with value of privacy (the value inconsistent with the proposed law). Thus, expanding the results of Study 1, Study 2 examined whether the motivation for the values underlying a law would be associated with intended law compliance. We pre-registered the following studies based on the results of Study 1, and keeping in mind the law literature, which suggests consequences are key to law compliance (Tao, 1976).

The following hypotheses were registered in advance of the study (https://osf.io/zwdf5):

### Hypothesis 1

Those higher on autonomous or controlled motivation to follow the proposed law would have higher intent to follow the proposed law.

### Hypothesis 2

Those higher on inclusion value internalization (who report more autonomous motivation and less controlled motivation for the value of inclusion) and lower on privacy value internalization (who report less autonomous motivation and more controlled motivation for the value of privacy) would have higher intent to follow the proposed law.

### Method

#### Participants

Sample size calculations (Faul et al., 2009) suggested that to achieve a power of.90, with a small effect size of.15, alpha error probability of.05, and ten predictors, we required a minimum of 147 participants. We recruited *N* = 217 healthcare professionals via Prolific.com to allow for exclusions. Of these, 66 were excluded for failing our stringent attention check (described below), and one was excluded because they did not work in human healthcare. The remaining *N* = 150 participants (106 women; 44 men) had a mean age of 32.2 years (*SD* = 10.18, range = 18–71 years). The sample was predominantly White (62.67%), with 14% Asian, 13.33% Black or African American, and 10% “Other”.

#### Materials

Measures were identical to Study 1 (autonomous motivation to follow the law, controlled motivation to follow the law, having a value of privacy, having a value of inclusion, intended law compliance), though four additional predictor variables were new to Study 2 (autonomous and controlled motivations for the *inclusion* value, autonomous and controlled motivations for the *privacy* value). Scales used in Study 2 showed high internal reliability: Autonomous (McDonald’s ω =.86, 95% CI [.80,.89]) and controlled (*r* =.45, *p* <.001) motivation.

*Internalization of inclusion and privacy values*: In addition to asking participants *whether* they value inclusion and privacy, they were also asked questions about their *motivation* for valuing inclusion and privacy. Participants were asked to complete the questionnaire on a 0 (“*strongly disagree”*) to 100 (“*strongly agree”*) scale assessing their reasons for following the law. Questions were created to mirror those measuring reasons to follow the law. For example, when measuring the inclusion value, autonomous motivation items were: “Because inclusion is in line with who I am”, “Because the ways that inclusion benefits society are important to me”, and “Because the value of inclusion is consistent with who I am”. Controlled motivation was measured with: "Because I would be judged if I didn't care about inclusion”, “Because I care about my reputation being damaged if I appear not to care”, and “Because I feel I have no choice but to act in line with the value of inclusion”. Motivation for having a privacy value was measured similarly to inclusion but replacing the value within the items. The measures showed high internal reliability: McDonald’s ω =.85, 95% CI [.80,.89] for autonomous inclusion motivation; *r* =.76, *p* <.001 for controlled inclusion motivation; McDonald’s ω =.85, 95% CI [.80,.89] for autonomous privacy motivation and *r* =.65, *p* <.001 for controlled privacy motivation.

#### Deviations from pre-registration

The study was pre-registered, but several deviations were made. First, our registration indicated that we included ten predictors to the model, but we ultimately selected not to include covariates (age, gender, ethnicity) in the final analysis because there was no theoretical justification for doing so. Amotivation for following the law was also pre-registered as a predictor but not included in the models because we selected to maintain focus on controlled versus autonomous motivations. Finally, internalization of inclusion and privacy, which was defined in pre-registration as “internalization = autonomous motivation– controlled motivation– amotivation” was not included in the model because it was collinear with autonomous and controlled motivations that were included in the models. The associations between our independent variables and the outcome remained the same when controlling for demographics and amotivation.

### Results

#### Analytic strategy

Planned analyses testing Hypotheses 1–3 remained the same as in Study 1, with the addition of four variables introduced in Study 2 (autonomous and controlled motivation for the values of inclusion and privacy).

Intended law compliance positively correlated with autonomous motivation to follow the law (*r* =.60, *p* <.001, large effect size), controlled motivation to follow the law (*r* =.18, *p* <.05, small effect size), and autonomous motivation for inclusion (*r* =.31, *p* <.001, moderate effect size). Autonomous motivation to follow the law was significantly positively correlated with valuing inclusion (*r* =.17, *p* <.05, small effect size), autonomous motivation for inclusion (*r* =.24, *p* <.01, small effect size), and controlled motivation for privacy (*r* =.46, *p* <.001, moderate effect size). Autonomous motivation to follow the law was also negatively correlated with valuing privacy (*r* =  −.17, *p* <.05, small effect size).

Controlled motivation to follow the law was positively correlated with controlled motivation for inclusion (*r* =.46, *p* <.001, moderate effect size). Valuing inclusion was positively correlated with autonomous motivation for inclusion (*r* =.60, *p* <.001, large effect size) and negatively correlated with controlled motivation for inclusion (*r* =  −.18, *p* <.05, small effect size). Autonomous motivation for privacy was related to valuing privacy (*r* =.73, *p* <.001, large effect size). See Table 3 for full details on correlation coefficients.

**Table 3:** Study 2 correlations between key variables

	1	2	3	4	5	6	7	8
1. Intended compliance	–							
2. Autonomy for law	.60***	–						
3. Control for law	.18*	−.14	–					
4. Valuing inclusion	.15	.17*	−.03	–				
5. Autonomy for inclusion	.31***	.24**	.04	.60***	–			
6. Control for inclusion	.06	.10	.46	−.18*	−.14	–		
7. Autonomy for privacy	−.01	−.13	−.02	.10	.13	.11	–	
8. Control for privacy	.09	.17*	.45	−.02	−.03	.61	.05	–
9. Valuing privacy	−.02	−.17*	−.04	.14	.07	.00	.73***	−.08

*p <.05, **p <.01, ***p <.001. Autonomy = autonomous motivation (variables 2, 5, 7); Control = controlled motivation (variables 3, 6, 8)

The full model indicated that autonomous and controlled motivation to follow the proposed law, and controlled motivation for inclusion were positively associated with intended law compliance. Having the values of inclusion and privacy, as well as autonomous and controlled motivation to act in line with values of inclusion and privacy were not associated with intended law compliance (see Table 4 for results of the full model). The reduced model, which only included autonomous and controlled motivation to follow the proposed law, as well as autonomous motivation for inclusion, as predictors, showed that those higher on autonomous motivation (*b* =.19, *SE* =.02, *p* <.001) and controlled motivation (*b* =.11, *SE* =.03, *p* <.001) to follow the law had greater intent to follow the law. Further, higher autonomous motivation for inclusion was associated with intended law compliance (*b* =.08, *SE* =.03, *p* =.030).

**Table 4:** Study 2 predictors of intended law compliance from the full model

Model		*b*	*SE*	β	*t*	*p*	95% CI	
							Lower	Upper
H_0_	(Intercept)	66.28	2.21		29.95	<.001	61.91	70.66
H_1_	(Intercept)	.30	12.96		.02	.981	− 25.31	25.92
	Intended compliance	.21	.02	.61	9.30	<.001	.16	.25
	Autonomy for law	.15	.03	.31	4.33	<.001	.08	.22
	Control for law	.10	.04	.20	2.53	.013	.02	.17
	Valuing inclusion	−.05	.04	−.12	− 1.42	.159	−.13	.02
	Autonomy for inclusion	−.18	.14	−.10	− 1.29	.200	−.46	.10
	Control for inclusion	.01	.04	.02	.22	.826	−.07	.09
	Autonomy for privacy	−.02	.04	−.05	−.62	.539	−.10	.05
	Control for privacy	.11	.14	.07	.79	.428	−.17	.40

### Brief discussion

Study 2 results showed that higher autonomous *and* higher controlled motivation to follow the law were related with higher intended law compliance, supporting Hypothesis 1 and literatures on rule and law compliance (Fast & Schnurr, 2021; Ryan & Deci, 2017). This is partially consistent with results of Study 1, which found that autonomous, but not controlled, motivation to follow the law was associated with intended law compliance. The discrepancy implies that while internal drive (autonomous motivation) consistently predicts compliance, the influence of external pressures (controlled motivation) may have variable effects and is in line with some previous applications of SDT, which have demonstrated consistent positive effects of autonomous motivation, but indicated mixed effects for controlled motivation (e.g., Kuvaas et al., 2017; Malhotra et al., 2008).

Different findings across the two studies may lie in the expanded design of Study 2. Unlike Study 1, Study 2 controlled for participants’ autonomous and controlled motivation to act in line with the value of inclusion (which aligns with the proposed law) and privacy (which stands in contrast to the law). It is possible that accounting for these factors helped clarify the unique contribution of controlled motivation to comply with the law itself. In Study 1, by contrast, the effect of controlled motivation may have been obscured by unmeasured individual differences in the extent to which participants felt internally or externally pushed to act on these underlying values.

Examining motivation for the values underlying laws, we found that greater autonomous, but not controlled, motivation for the value of inclusion was related with intended law compliance. This partially supported Hypothesis 2 and suggests that motivation to act in line with values of inclusion that comes from within individual could be associated with behavioral intention. As was the case in Study 1, endorsement and motivation to act in line with the value of privacy– the value that stood in contrast with the proposed law– were not related to the outcome. Across both studies, the findings suggest that law compliance is more strongly motivated by alignment between laws and individuals’ values, rather than by laws that conflict with those values. This distinction is important for future research seeking to understand why people may support proposed laws that, despite appearing problematic, resonate with or reinforce their existing value structures (Tyler, 2006).

## Study 3

Studies 1–2 demonstrated that both autonomous and controlled motivation contribute to legal compliance, with autonomous motivation being a particularly strong predictor. Building on these findings, Study 3 introduces an experimental component to test whether the severity of legal consequences influences compliance intentions and how these consequences interact with motivation. Further, based on pilot data for this study (see supplementary materials), we have introduced individuals’ *general* motivation to follow laws. If people with high autonomous motivation for laws in general are simply more likely to comply with laws, then the effects of value-based motivation may be overestimated.

Classic deterrence theory posits that individuals comply with laws due to the fear of punishment (Beccaria, 1764/2017), whereas SDT perspective suggests that intrinsic motivation may foster more enduring compliance (Deci & Ryan, 1985). However, to our knowledge, no research has experimentally tested how different levels of legal consequences interact with motivation to shape compliance intentions.

Intended compliance is associated with Study 3 utilized an experimental design to examine how intended compliance varies based on the severity of consequences for breaking the law. Participants were presented with three levels of severity: mild (a warning), moderate (a $17,500 fine), and severe (revocation of a clinician’s license). The following hypotheses were tested and pre-registered (https://osf.io/5e47h):

### Hypothesis 1

Higher autonomous motivation to follow the law would be associated with greater intended law compliance.

### Hypothesis 2

More severe consequences for breaking the law would be associated with higher intended law compliance.

### Method

#### Participants

We recruited *N* = 298 healthcare professionals. Of these, 61 were excluded for failing the attention check (described below). The remaining *N* = 237 participants (161 women; 73 men; 2 non-binary; 1 preferred not to disclose) had a mean age of 34.21 years (*SD* = 11.44, range = 18–75 years). The sample was predominantly White (59%), with 15% being Black or African American, 15% Asian, and 10% “Other”. Participants were recruited from Prolific without screening for prior participation in related studies. Therefore, there is a possibility of a sample overlap. However, given the experimental nature of the study, we expected that participants’ responses would not be biased by prior participation.

#### Materials

This study utilized the same measures as were used in previous studies. Namely, we once again measured autonomous motivation to follow the law (ω =.84, 95% CI [.81,.88]), controlled motivation to follow the law (*r* =.38, *p* <.001), having a value of privacy, having a value of inclusion, autonomous motivation for inclusion (ω =.92, 95% CI [.91,.94]), controlled motivation for inclusion (*r* =.73, *p* <.001), autonomous motivation for privacy (ω =.88, 95% CI [.86,.91]), controlled motivation for privacy (*r* =.71, *p* <.001), and intended law compliance, with the addition of “motivation to cooperate with the law in general” predictor variable. In addition, the study involved an experimental condition.

*Motivation to Cooperate with the Law in General*: Before asking participants to read the law, we assessed their reasons for following the law in general. Participants were asked to complete the questionnaire on a scale from 0 (“*strongly disagree”*) to 100 (“*strongly agree”*). Items were paired with a stem asking: “What are your reasons for obeying the law?” Autonomous motivation was measured with the following items: “because following the law is in line with who I am”, “because the ways that law benefits society are important to me”, and “because obeying the law is in line with who I am” (ω =.89, 95% CI [.86,.91]). Controlled motivation was measured with: “because I would be punished if I didn’t follow the law”, “because I care about my reputation being damaged if I don’t follow the law”, and “because I feel I have no choice but to follow the law” (*r* =.41, *p* <.001).

*Condition*: We piloted the study with 30 clinicians to identify which consequences would be considered mild, moderate, and severe. In the main study, depending on assignment to random condition, participants were also exposed to receiving one of the following statements concerning consequences for failure to comply with the law: *Condition 1*) mild consequences: Failure to comply with the law will result in clinicians receiving a warning. *Condition 2*) moderate consequences: Failure to comply with the law will result in a fine of 17,500 USD. *Condition 3)* severe *consequences*: Failure to comply with the law will result in clinicians losing their license.

As a manipulation check, participants were asked to summarize the proposed law and consequences in their own words.

*Perceived severity of consequences* The manipulation check was conducted by asking participants what the consequence is for breaking the law and how severe they believed the penalty to be on a scale from 0– “*very mild*” to 100– “*very severe*”.

*Perceived appropriateness of consequences* We also asked participants to rate their agreement with the statement “I believe the penalty for breaking this law is appropriate” on a scale from 0– “*definitely not*” to 100– “*definitely yes*”.

#### Deviations from pre-registration

In the analytic strategy that we pre-registered, we indicated that we would consider penalty for breaking the law in a separate model. However, as we believe that it is important to control for values in the same model, we included all predictors within one model. Results testing our primary predictors separately showed similar effects in terms of direction and significance.

#### Analytic strategy

The analytic strategy followed the approach we took in Study 2. However, in the current study, consequence severity was added to each model as predictors. Further, in each model, we controlled for the perceived appropriateness of the consequences.

### Results

Intended law compliance was correlated with autonomous motivation to follow the law (*r* =.56, *p* <.001, large effect size), controlled motivation to follow the law (*r* =.43, *p* <.001, moderate effect size), controlled motivation for inclusion (*r* =.22, *p* <.001, small effect size), valuing inclusion (*r* =.18, *p* <.01, small effect size), controlled motivation for privacy (*r* =.13, *p* <.05, small effect size), autonomous motivation to cooperate with law (*r* =.21, *p* <.01, small effect size), controlled motivation to cooperate with law (*r* =.19, *p* <.01, small effect size), and perception that penalty is appropriate (*r* =.39, *p* <.001, moderate effect size).

Autonomous motivation to follow the law was correlated with controlled motivation to follow the law (*r* =.48, *p* <.001, moderate effect size), autonomous motivation for inclusion (*r* =.21, *p* <.001, small effect size), controlled motivation for inclusion (*r* =.41, *p* <.001, moderate effect size), valuing inclusion (*r* =.25, *p* <.001, small effect size), controlled motivation for privacy (*r* =.31, *p* <.001, moderate effect size), autonomous motivation to cooperate with law (*r* =.30, *p* <.001, moderate effect size), controlled motivation to cooperate with law (*r* =.32, *p* <.001, moderate effect size), and perception that penalty is appropriate (*r* =.56, *p* <.001, large effect size).

Controlled motivation to follow the law was correlated with controlled motivation for inclusion (*r* =.39, *p* <.001, moderate effect size), controlled motivation for privacy (*r* =.32, *p* <.001, moderate effect size), autonomous motivation to cooperate with law (*r* =.23, *p* <.001, small effect size), and controlled motivation to cooperate with law (*r* =.47, *p* <.001, large effect size).

Autonomous motivation for inclusion was correlated with valuing inclusion (*r* =.81, *p* <.001, large effect size), autonomous motivation for privacy (*r* =.17, *p* <.01, small effect size), and autonomous motivation to cooperate with law (*r* =.36, *p* <.001, moderate effect size).

Controlled motivation for inclusion was correlated with controlled motivation for privacy (*r* =.69, *p* <.001, large effect size), controlled motivation to cooperate with law (*r* =.39, *p* <.001, moderate effect size), and perception that penalty is appropriate (*r* =.17, *p* <.01, small effect size).

Valuing inclusion was correlated with autonomous motivation to cooperate with law (*r* =.31, *p* <.001, moderate effect size).

Autonomous motivation for privacy was positively correlated with valuing privacy (*r* =.69, *p* <.001, large effect size) and autonomous motivation to cooperate with law (*r* =.34, *p* <.001, moderate effect size).

Controlled motivation for privacy was positively correlated with controlled motivation to cooperate with law (*r* =.36, *p* <.001, moderate effect size).

Valuing privacy was correlated with autonomous motivation to cooperate with law (*r* =.22, *p* <.001, small effect size).

Autonomous motivation to cooperate with law was correlated with controlled motivation to cooperate with law (*r* =.30, *p* <.001, moderate effect size) and perception that penalty is appropriate (*r* =.20, *p* <.01, small effect size).

Penalty severity condition was negatively correlated with autonomous motivation to follow the law (*r* =  −.14, *p* <.05, small effect size), valuing inclusion (*r* =  −.14, *p* <.05, small effect size), and perception that penalty is appropriate (*r* =  −.32, *p* <.001, moderate effect size).

The perception that penalty is appropriate was positively correlated with controlled motivation for inclusion (*r* =.17, *p* <.01, small effect size) and autonomous motivation for inclusion (*r* =.16,* p* <.05, small effect size).

See Table 5 for full details.

**Table 5:** Study 3 correlations between key variables

	1	2	3	4	5	6	7	8	9	10	11	12
1. Intended law compliance	–											
2. Autonomy for law	.56***	–										
3. Control for the law	.43***	.48***	–									
4. Autonomy for inclusion	.15*	.21***	.10	–								
5. Control for inclusion	.22***	.41***	.39***	−.14*	–							
6. Valuing inclusion	.18**	.25***	.10	.81***	−.12	–						
7. Autonomy for privacy	−.06	−.03	.12	.17**	−.05	.10	–					
8. Control for privacy	.13*	.31***	.32***	−.09	.69***	−.07	−.10	–				
9. Valuing privacy	−.03	−.07	.16*	.09	.01	.08	.69***	−.04	–			
10. Autonomy to cooperate with law in general	.21**	.30***	.23***	.36***	.13*	.31***	.34***	.07	.22***	–		
11. Control to cooperate with law in general	.19**	.32***	.47***	.13*	.39***	.14*	.18**	.36***	.16*	.30***	–	
12. Penalty severity condition	.04	−.14*	.13*	−.08	.01	−.14*	−.10	.00	<.001	−.07	−.03	–
13. Penalty as appropriate	.39***	.56***	.13	.16*	.17**	.16*	−.04	.11	−.11	.20**	.04	−.32***

*p <.05, **p <.01, ***p <.001. Condition coded as 1—mild, 2—moderate, 3—severe. Autonomy = autonomous motivation (variables 2, 4, 7, 10); Control = controlled motivation (variables 3, 5, 8, 11)

Results for the full model can be found in Table 6. The reduced model, which only included autonomous motivation to follow the law, controlled motivation to follow the law, perception that penalty is appropriate, and punishment severity (condition) as predictors, showed that those higher on autonomous motivation (*b* =.13, *SE* =.03, *p* <.001) and controlled motivation (*b* =.11, *SE* =.03, *p* <.001) to follow the law had greater compliance intention. Higher perception that the penalty was appropriate (*b* =.16, *SE* =.06, *p* <.001) and higher punishment severity (*b* = 4.30, *SE* =.05, *p* =.020) were associated with higher intended law compliance.

**Table 6:** Study 3 predictors of intended law compliance from the full model

Model		*b*	*SE*	β	*t*	*p*	95% CI	
							Lower	Upper
H_0_	(Intercept)	68.84	1.78		38.64	<.001	65.33	72.35
H_1_	(Intercept)	19.99	1129		1.77	.069	− 2.26	42.24
	Autonomy for law	.13	.03	.37	4.74	<.001	.08	.19
	Control for law	.13	.04	.24	3.51	<.001	.06	.21
	Autonomy for inclusion	−.02	.04	−.04	−.40	.807	−.10	.07
	Control for inclusion	.00	.04	.00	.04	.945	−.07	.07
	Having inclusion value	.10	.13	.07	0.81	.546	−.15	.35
	Autonomy for privacy	−.05	.04	−.11	− 1.46	.109	−.13	.02
	Control for privacy	−.04	.03	−.09	− 1.19	.235	−.10	.03
	Having privacy value	.07	.12	.04	0.59	.517	−.16	.30
	Autonomy to cooperate with law in general	.02	.03	.05	0.83	.423	−.03	.08
	Control to cooperate with law in general	−.02	.04	−.02	−.37	.679	−.10	.07
	Penalty severity condition	4.01	.92	.12	2.08	.002	.22	7.80
	Perception that penalty is appropriate	.15	.06	.18	2.74	.017	.04	.26

Condition coded as 1 — mild, 2 — moderate, 3 — severe

### Brief discussion

Study 3 results indicated that penalty severity was associated with greater intended law compliance. Hypothesis 2 was therefore partially supported. This finding suggested that individuals may be more inclined to comply with legal norms when the penalties for non-compliance are more severe, consistent with previous research indicating that severity of punishment could affect law compliance (Karper & Lopes, 2014). Consistently with hypotheses and the results of previous studies, autonomous motivation to follow the law was associated with greater intended law compliance. Further, in this study, controlled motivation was again linked to *more* intended law compliance, suggesting that while control-motivated individuals may harbor negative attitudes towards the law, it may still be associated with increased compliance.^1^

## General discussion

Across three studies, the present research integrated SDT with perspectives from the field of law to inform and expand our understanding of which motivational factors are associated with intended compliance with laws. To stretch understanding and apply the question of legal compliance to an important context where new laws are much needed, we explored these processes in the context of healthcare laws, where two key values of privacy (i.e., data privacy) and inclusion (i.e., inclusive, fair technology development) have lived in tension (Whittlestone et al., 2019). Considering psychological principles alongside expectations from the legal field, we examined the relative predictive power of both autonomous and controlled motivation for following a law. Furthermore, given the close links between laws and the values underlying them, we explored the two values most relevant to our proposed law: namely, inclusion– a value underlying the proposed law, and privacy, a value that is in tension with the proposed law.

### Theoretical implications

Our studies were concerned with motivational factors underlying intended compliance, an important outcome in the context of law construction. Laws, though crucial for societal order, are rendered ineffective without compliance (Levi et al., 2012). Findings for intended compliance were consistent with our expectations based on SDT: those who had greater autonomous motivation for a law reported greater compliance intention. These results were held even when reporting holding the values underlying the proposed law (Studies 1–3), accounting for autonomous motivation to follow laws, in general (Study 3) and severity of the consequences of failure to follow the law (Study 3). Our findings highlighting consistent benefits of autonomous motivation to law compliance were consistent with those of Van Petegem et al. (2021), who found that autonomous, not controlled motivation predicts adolescents’ compliance with the law, with Sanderson and Darley (2002), who showed that individuals believe that they obey the law mostly for internal (e.g., values), rather than external reasons (e.g., punishment), and with work by Martela et al. (2021), who showed that using autonomy-supportive language is associated with autonomous forms of compliance.

Building on this extant research, the current studies integrated the SDT and the legal perspectives and provided evidence to support the role of autonomous motivation in driving invested (clinicians’) intentions to comply with proposed legal mandates that would affect their mode of care in ways that align or conflict with their values. This advancement is important because in real-world contexts, values may be in tension with imposed laws. Our findings therefore offer guidance for policymakers and legal drafters: to cultivate genuine compliance and encourage meaningful, invested engagement, laws should be aligned with existing societal values and communicated in ways that clearly highlight the values they aim to uphold.

Yet, in contrast to certain of life’s contexts where autonomous motivation is singularly beneficial (e.g., Weinstein 2014), we suspected that controlled motivation might be a predictor of greater compliance intention based on dominant perspectives within the field of law (Kahan, 1999; Nagin, 1998; Simpson et al., 2013). Evidence in support of this view was mixed across studies. Whereas controlled motivation independently contributed to intended compliance in Studies 2 and 3, it showed no benefits (but also no costs) for intention to comply with the law in Study 1. It is possible that the modified design and analytic strategy of Studies 2 and 3 contributed to emerging controlled motivation as a significant predictor. As such, within these studies, we included the measure of whether the motivation for values themselves was autonomous or controlled. The current results contributed to a growing body of research that suggests inconclusive effects of controlled motivation on behavior intention. While some studies find that controlled motivation could be beneficial (Fast & Schnurr, 2021), others find null results (Koestner et al., 2008), or even negative relations to behavior (Gaudreau et al., 2012; Tam et al., 2019). In the context of compliance with laws, it appears that controlled motivation driven by social and internalized pressures could potentially contribute to its effectiveness, but unlike autonomous motivation, its benefits are unstable. Future research that explores the boundary conditions of controlled motivation where it may no longer benefit compliance may challenge the existing status quo in the legal field. Regardless, the current findings suggest that the power of autonomous motivation should not be overlooked by law- and policy-makers who aim to drive societal change through regulation. The current studies highlight that compliance is maximized when the public can come to place personal value and meaning on proposed laws.

In all, the results of the current research suggest that promoting autonomous motivation when enacting and communicating about new laws may ultimately be more effective than a singular focus on the punishment. Together with the findings of, our findings suggested that promoting autonomous motivation is the best way to ensure law compliance.

### Future directions and applications

Our studies tested not only internalized law motivation broadly, but also the internalization of specific values underlying law to examine complex drivers of law compliance that may operate on behavior in the real world. This approach distinguished between simply having a value and internalizing the value such that it is felt to be personally valuable to the self rather than being externally imposed. Previous research has pointed in this direction when testing autonomous motivation that is underlying value-expressing behaviors such as blood donations (Williams et al., 2019), pro-environmental actions (Kaplan & Madjar, 2015), and multiple health behaviors (Hagger et al., 2014; Morell-Gomis et al., 2018). The current findings alongside previous research suggest that autonomous motivation underlying values may encourage individuals to engage with them. Future research identifying specific conditions under which values are more or less likely to be internalized, particularly in contexts where laws are contested or socially divisive, is important for understanding compliance with value-driven policies. Doing so could add needed predictive power to understand when laws are likely to be perceived as legitimate and personally meaningful, ultimately enhancing compliance and public trust.

Another important consideration for future research is whether different laws interact in different ways with autonomous and controlled motivation. For example, laws that require proactive action (e.g., wearing a seatbelt, using designated pedestrian crossings) as opposed to laws that prohibit certain behaviors (e.g., running a red light, exceeding speed limits) may have different relationships to reasons for compliance. It is plausible that in these contexts, controlled motivation may promote short-term obedience in high-risk situations (e.g., crossing the busy road) but fail to sustain ongoing compliance once the external pressure is removed (e.g., crossing the empty road). Future research could explore whether motivation types differentially predict compliance depending on whether a law requires action or restraint, further refining our understanding of legal internalization and compliance.

In addition, the research findings speak to the field of law, and we hope they might be a starting point for a more nuanced perspective of how motivators drive enforcement of new legal frameworks, including the provisional agreement on a European Health Data Space (EHDS; European Parliament, 2024). The EHDS provides a new and permissive framework on the secondary use of health data and data-driven innovation in healthcare (Hussein et al., 2023). The results of this research can inform future directions in leveraging health data for societal benefit, while maintaining privacy and prompting inclusion. This might speak to a more contextual approach when looking at the role of societal interests and public benefit with regard to such regulations as those concerning data sharing (Staunton et al., 2024). Future studies could continue to expand the applications of the SDT to a legal field and focus on the interactions between perceived autonomy and perceived institutional legitimacy (Jackson et al., 2012).

It is worth noting that the dynamics studied here — along with others to be explored in future work — exist within broader cultural and social systems that shape them. Further research is needed to explore these important contexts for law motivation. For example, though not tested here, social norms are important drivers of behavior that can influence both motivation and compliance (Amiot et al., 2013; Chatzisarantis & Biddle, 1998; Grayson et al., 2019). In the context of healthcare laws, motivation and behavior might be influenced by the extent that colleagues of clinicians consistently obey or disobey the law in question, regardless of whether the law is personally meaningful to them (see MacIntyre, 2005).

### Limitations of the research

The results of the current series of studies should be considered in the context of the limitations. First, although Study 3 experimentally manipulated consequences, our primary findings were based on correlational data reporting motivation towards and views regarding laws. Further studies could examine both motivational and law principles using experimental designs, for example by exposing participants to different proposed laws, including condition that prioritizes one value, such as inclusion, over a value in tension, such as privacy. Studies could also manipulate the framing of the law exposure– emphasizing the benefit of the proposed law and downplaying potential negative effects versus emphasizing both the value it helps to uphold *and* the value that it sacrifices. Given the importance of autonomous motivation for attitudes and intention concerned with the law in the current studies, future studies that manipulate the framing of laws in autonomy-supportive or controlling ways will provide important avenues to further explore motivational pathways for law compliance, building on the body of work on public health messages that manipulate motivational framing (Pelletier & Sharp, 2008). Such framing may itself be difficult to effectively implement but offers great social benefit if delivered effectively (Legate et al., 2022).

Second, our study relied on self-report method of data collection. However, people tend to attribute their reasons for following the law to internal factors (such as morality and respect for law), while also tending to believe that those who previously broke the law follow the law because of external factors (e.g., fear of punishment; Sanderson & Darley, 2002). Therefore, a self-serving bias could be in play here and could offer an alternative explanation for our results suggesting that autonomous motivation was key in driving intended compliance. To extend the current results, future studies could utilize other indicators, such as through analysis of social media content (Lai & To, 2015) and examine views on laws and compliance intention. In doing so, future research can consider actual laws as compared to the proposed law we utilized in this research and examine behavioral rather than focusing on behavioral intention. Such research will bring needed ecological validity to this area.

Third, in Study 3, participants were recruited from Prolific without screening for prior participation in related studies. Therefore, there is a possibility of sample overlap. However, given the experimental nature of the study, we expected that participants’ responses would not be biased by prior participation. Further, it is important to acknowledge the limitation of the measurement of motivation, particularly the wording of certain items within this scale. While our measure was designed to align with established SDT principles, the item ‘Because I believe this law is fair, just, and legitimate’ may not fully capture autonomous motivation in a way that is independent of individual differences in valuing fairness, justice, and legitimacy. The current research does not aim to serve as a final scale development effort. Instead, we view it as an important step toward refining how SDT is applied to the study of legal motivation. Indeed, despite the preliminary nature of scale development in these studies, the two items for controlled motivation demonstrated a correlation of *r* >.40, suggesting an acceptable level of internal consistency. Nonetheless, future research should further develop and validate motivation measures to ensure more precise distinctions between identified and integrated forms of autonomous motivation, as well as between controlled motivation and amotivation. Such refinements will contribute greatly to making robust theoretically informed conclusions based on SDT in legal and policy contexts.

Finally, the present research did not directly assess participants’ perceptions of the values underpinning the proposed law. While participants were asked about their reasons for following the law, including options such as “Because inclusion is in line with who I am”, we did not explicitly measure whether participants themselves viewed the law as grounded in inclusion or as compromising privacy. The study design assumes that participants perceive the law’s purpose (i.e., to reduce algorithmic bias) as reflecting the value of inclusion, and its data-sharing provisions as potentially undermining privacy. However, individual interpretations of legal values can vary, and some participants may not have construed bias reduction as an inclusion-related goal. This disconnect between the law’s intended foundation and its perceived foundation could influence the strength of the relationship between value-based motivation and intention to comply. Future research should assess perceived value alignment more directly to better capture how individuals interpret and internalize the ethical foundations of legal mandates.

## Conclusions

This series of studies integrated SDT views with those dominant within the legal field, and by doing so offered a novel lens through which legal compliance could be examined. Results showed a consistent pattern wherein autonomous motivation to follow the law was the main driver in intention to comply with the law. In contrast, the positive association between controlled motivation and intended law compliance was not consistently supported. In all, autonomous, rather than controlled motivation was shown to be the key in ensuring intention to follow the law. Our results show promise in the application of SDT to the broader fields beyond psychology. Therefore, we encourage future research to explore the role of autonomous motivation in broader societal-level governance issues, encouraging further cross-pollination between SDT and fields such as criminology, policy, and legal psychology.

## Footnotes

1 Please refer to the supplementary analysis for the factor analysis for Studies 1-3, for results of pilot data for Study 3, exploratory mediation analyses, and the results of two additional outcomes: law favorability and law support, across all studies.
